# Editorial: Violence and mental health. focus on schizophrenia spectrum and psychotic disorders

**DOI:** 10.3389/fpsyt.2025.1618000

**Published:** 2025-05-08

**Authors:** Massimo Tusconi, Serdar M. Dursun

**Affiliations:** ^1^ Department of Medical Sciences and Public Health, University of Cagliari, Cagliari, Italy; ^2^ Neurochemical Research Unit, Department of Psychiatry University of Alberta, Edmonton, AB, Canada

**Keywords:** biomarkers, neurobiology, neuroimaging, schizophrenia, violence, recovery, psychosis

In recent years, the intersection of violence and mental health has become an increasingly debated topic, often characterized by controversy and misunderstanding (1, 2). Among the psychiatric conditions most frequently associated, often erroneously, with violent behavior, the spectrum of schizophrenia and other psychotic disorders stands out (3–5). This association, however, is not only simplistic and stigmatizing but also dangerous, as it distorts public perception and undermines the dignity and rights of people living with mental illness (6). The scientific evidence indicates that the vast majority of people with schizophrenia or other psychotic spectrum disorders are not violent (7–10). They are much more likely to be victims of violence than perpetrators (11, 12). Media narratives, which often sensationalize rare and extreme cases, contribute to distorting the image of mental illness as inherently dangerous (13, 14); this not only exacerbates stigma but also fuels fear and discrimination, making it more difficult for people to seek help and receive adequate care. Violence is a complex and multifactorial phenomenon influenced by biological, psychological, and social factors (15, 16). The influence of comorbid conditions and states initially diagnosed as bipolar, which have changed over time due to our increased knowledge of modifications in brain and molecular structure, also emerges in diagnoses of the psychotic spectrum (17–24). While some symptoms, such as persecutory delusions or grandiose hallucinations, may be associated with an increased risk of violent behavior in some individuals, these cases are the exception, not the rule. However, these symptoms are sometimes used as a justification for violent actions, which, far from being attributable in a robust and irrefutable way to psychotic symptoms, end up increasing the social stigma towards these pathologies even more (25, 26). Moreover, concomitant factors, such as substance abuse, socioeconomic deprivation, the lack of protective factors, and exposure to trauma play a much more substantial role in the emergence of violent behavior, whether directed against oneself or others, than the mere presence of a psychotic disorder (27–29). It is essential to dispel the myth that mental illness equals violence. To do so requires a critical examination of the scientific data and a cultural shift that promotes empathy, inclusion, and evidence-based communication (30–32). Mental illness should never be used as a scapegoat for criminal actions (33). Hopefully, legal and law systems should avoid generalizations and ensure that psychiatric diagnoses are not misused to justify or explain criminal behavior without a nuanced and personalized assessment (14, 34, 35).

Regarding empathy deficit in male patients with schizophrenia and its relationship with impulsivity and premeditated violence, Gong et al. report in their study that scores related to premeditated aggression are linked to aggressive behavior in patients with schizophrenia, establishing that the trait of premeditated aggression in these patients is a significant predictor of violent aggression. In their findings, patients with violent schizophrenia had more extensive empathy deficits than patients with non-violent schizophrenia, particularly in males. Further analysis also revealed that deficits in empathic ability in male patients with schizophrenia were strongly and positively correlated with premeditation characteristics, while they were not significantly correlated with impulsivity characteristics. These findings could be used in the future to predict the occurrence of premeditated aggression in male patients with schizophrenia through empathy assessments.

In their systematic review of machine learning (ML) for predicting violent behavior in schizophrenia spectrum disorders, Parsaei et al. describe the results of an in-depth evaluation of the literature in this field. They find that ML models have produced convincing results, highlighting the importance of their use in advanced diagnostics. The authors report that given the rapid growth in the application of various artificial intelligence tools in medical contexts, it seems likely that in the coming years ML models could also be used to predict violent behaviors in patients with schizophrenia spectrum disorders. These tools could be used for timely preventive interventions, such as providing social support and rehabilitation, adjusting medications, and considering more personalized therapeutic approaches, significantly reducing the burden of violent behavior on patients, healthcare systems, and society in general.

Additionally, in a randomized controlled trial, Li Z. et al. analyzed the neurofeedback technique for treating male patients with schizophrenia and impulsive behavior. By combining existing scientific evidence with the results of their study, the authors offer new insights and theoretical foundations for the treatment of impulsive behavior in male patients with schizophrenia, demonstrating that six weeks of systematic neurofeedback treatment significantly improves the severity of impulsive behaviors and reduces aggression in these patients.

Shifting the focus to network analysis of clinical characteristics in patients with treatment-resistant schizophrenia (TRS), the work of Li W. et al. describes treatment resistance in schizophrenia as multifactorial. At present, no single definition encompasses all aspects, as the pathogenesis is not well understood and the disease remains poorly characterized. From a symptomatic point of view, positive and negative symptoms are key clinical features of TRS; it also appears that differences in core symptoms between TRS and non-treatment-resistant schizophrenia may partly explain this particular resistance. The authors conclude that managing positive and negative symptoms in TRS remains crucial, with particular attention to negative symptoms and related clinical characteristics.

In an in-depth study of cognitive impairment and cortical thickness abnormalities in first-episode schizophrenia patients who had not previously been treated with medication and who exhibited symptoms of agitation, Liang et al. explored the relationships between agitated behavior, cognitive function, and cortical thickness in first-episode schizophrenia not treated with medication (FESN). Based on the results of their study, the authors report that working memory performed worse in FESN and agitation (FESN+A) patients than in controls; furthermore, cortical thickness of the left paracalcarine gyrus was increased in the FESN and non-agitation (FESN-NA) group compared to the healthy control group. The FESN+A group had greater cortical thickness in the right posterior cingulate cortex (rPCC) than the FESN+NA group. The cortical thickness of the rPCC was negatively correlated with working memory scores in the FESN+A group. The authors conclude that abnormal cortical thickness of the rPCC may be related to agitation behavior and cognitive function in patients with FESN+A, suggesting a potential therapeutic target for agitation behavior and cognitive impairment in schizophrenia.

Bravve et al. present a systematic review of suicide risk in patients with aggression in schizophrenia; in their assessment, the authors highlight that suicide is the leading risk factor for mortality among individuals with schizophrenia, with a mortality rate 10 times higher than the general population. In the study conducted on individuals who committed suicide, some showed a high risk of aggression and impulsivity, which allowed these indicators to be considered predictors of suicide risk.

Based on the evaluation of the proposed studies and the currently available literature, it is important not to minimize the complexity of psychotic disorders and the suffering involved with them (36–39). These conditions can profoundly affect a person’s perception of reality, emotional regulation, and social functioning (40, 41). Therefore, early intervention, integrated care, and social rehabilitation, by any means currently provided by scientific literature (37), including and not only modern applications of virtual reality for rehabilitation purposes, are fundamental to promoting recovery and reducing the risk of social marginalization, which in itself can contribute to increasing vulnerability (42–44). In conclusion, addressing the link between violence and mental health in the context of the spectrum of schizophrenia and psychotic disorders requires a balanced and unbiased approach (45, 46). We must strive to protect public safety without compromising the rights and dignity of people with mental illness. The way forward is a nuanced, compassionate understanding; the integration of such an approach with the most modern preventive and rehabilitative techniques and with pharmacological therapies could effectively enable the prevention of the most marked episodes of violence and the achievement of an increasingly optimal outcome.
